# Supplementary figures and images for: Genome-Wide Identification and Comparative Analysis of Myosin Gene Family in Four Major Cotton Species
Source: Genes (Basel). 2020 Jun 30;11(7):731. doi: 10.3390/genes11070731 (PMC7397272; doi:10.3390/genes11070731)

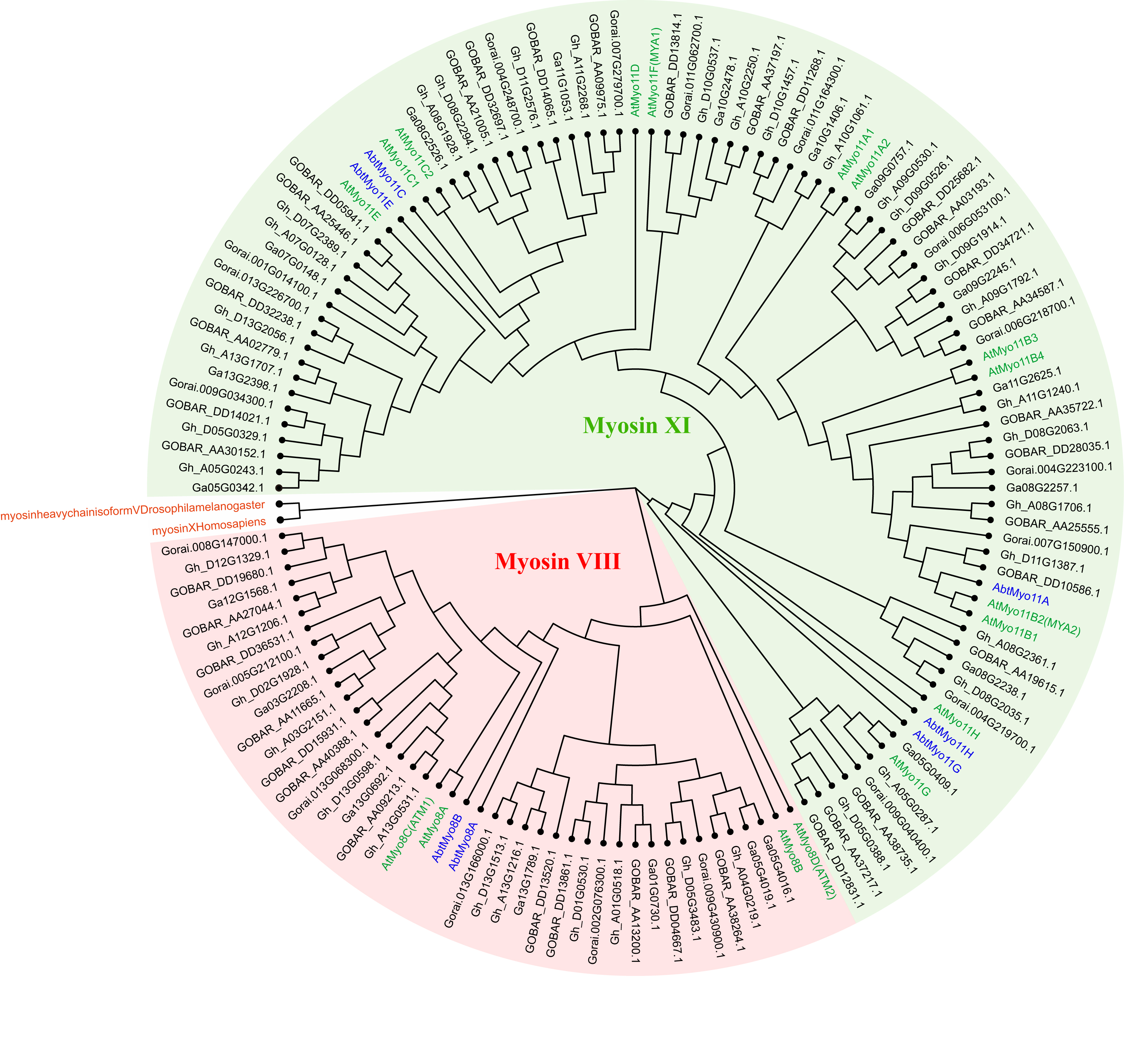

Supplement: Supplementary file 1 [file genes-11-00731-s001.zip › Supplement Material/Figure S1 Evolutionary tree.jpg]

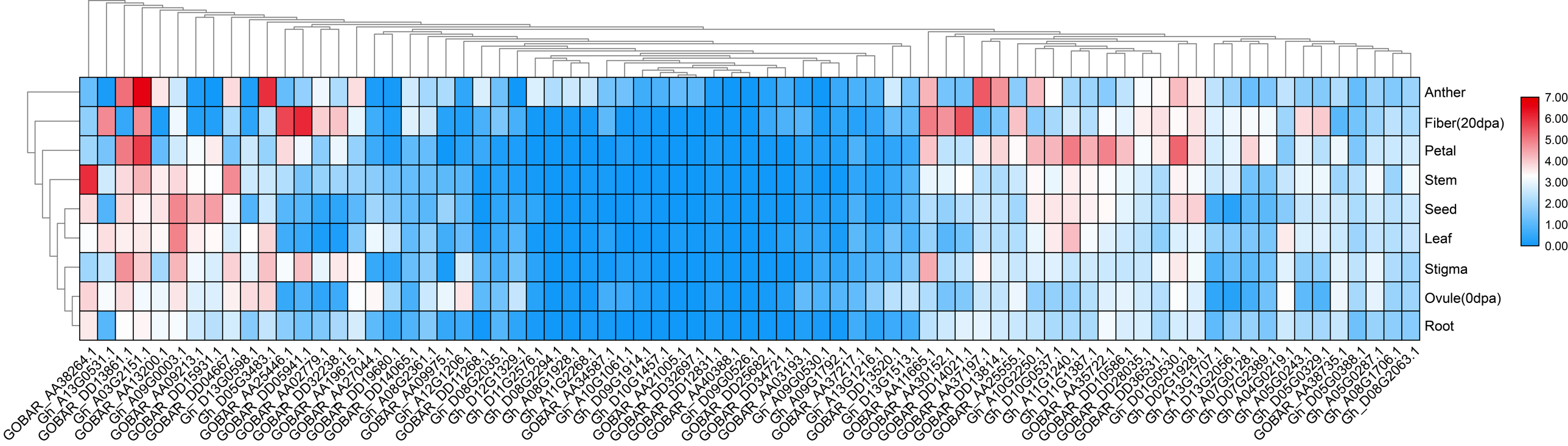

Supplement: Supplementary file 1 [file genes-11-00731-s001.zip › Supplement Material/Figure S2 Expression pattern.jpg]
